# Antibacterial activities of triterpenoidal compounds isolated from *Calothamnus quadrifidus* leaves

**DOI:** 10.1186/s12906-019-2512-x

**Published:** 2019-05-09

**Authors:** H. A. Ibrahim, M. R. Elgindi, R. R. Ibrahim, D. G. El-Hosari

**Affiliations:** Department of Pharmacognosy, Faculty of Pharmacy, Helwan University, EinHelwan, Cairo, 11795 Egypt

**Keywords:** Antibacterial, *Calothamnus quadrifidus*, Myrtaceae, Triterpene

## Abstract

**Background:**

*Calothamnus quadrifidus* R.Br has many traditional uses and there are few reports about its chemical and biological activities. So our aim is to isolate the triterpenoidal compounds from dichloromethane fraction (DCMF) of *Calothamnus quadrifidus* R.Br leaves and in addition to evaluate the antibacterial activity of the isolated compounds.

**Methods:**

DCMF of *C. quadrifidus* leaves was subjected to different chromatographic techniques to isolate pure triterpenoidal compounds which were identified using different chemical and spectroscopic techniques. Antibacterial activities of the isolated compounds were evaluated using agar well diffusion method while minimum inhibitory concentration was assessed by microtiter plate assay method.

**Results:**

Five compounds were isolated and they were betulinic acid (1), ursolic acid (2), 3-acetyl-23-hydroxy betulinic acid (3), 2,23-dihydroxy betulinic acid (4) and 2,21,23-trihydroxy betulinic acid (5) were isolated from DCMF of *C. quadrifidus* leaves for the first time. Compounds 4 and 5 showed strong antibacterial activity against *S. typhimurium* while compound 4, 5 and 3, 4 exhibits moderate effect against *E.coli* and *S. aureus* respectively.

**Conclusion:**

Pure triterpenoidal compounds isolated from *C. quadrifidus* leaves showed antibacterial activities in different strengths.

## Background

Genus *Calothamnus* (F. Myrtaceae) is commonly known as the one-sided bottle brush or a claw flower and comprises about 40 species. It is native to south Western Australia [1] and it is also cultivated in Egypt. *C. quadrifidus* R.Br is an erect or spreading shrub of about three meter high with red flowers arranged in clusters on one side of the stem [2]. Few reports concerning the phytoconstitutents of *C. quadrifidus*, mainly focused on phenolics, including flavonols, flavanones, flavones, tannins and phenolic acids [3, 4] as well as evaluation of its essential oil was reported [3]. Moreover it was found that the aqueous ethanol extract of the aerial parts and aqueous methanol extract of leaves and stems for *C. quadrifidus* possesses analgesic, anti-inflammatory, hypoglycaemic and antioxidant activities [3, 4]. In addition, the essential oil of the aerial parts showed antimicrobial activity [3]. There were no reports about investigation of the triterpenoidal content, so we deemed it of interest to isolate and identify the triterpene compounds from DCMF of *C. quadrifidus* leaves and to evaluate the antibacterial activity of the pure isolates.

## Methods

### Instruments and material

Silica gel-60 (Fluka Chemie AG, Switzerland) and sephadex LH-20 (Sigma-Aldrich Steinheim, Germany) were used as an adsorbent for column chromatography as well as pre-coated silica gel plates F_254_(Merck, Germany) were used for thin layer chromatography. *P*-anisaldehyde spray reagent was used for the detection of triterpenes. DCM/ MeOH; 95:5 (S_1_); 90:10 (S_2_) v/v, were used as solvent systems. The NMR data was measured using Bruker Avance (600 and 150 MHz for ^1^H and ^13^C NMR). Results were reported as δ ppm values relative to TMS as internal reference. The IR was carried out on FT/IR 300 E Jasco using KBr discs.

### Plant material

*C. quadrifidus* R.Br leaves were obtained from research park at Saft El-laban area, Giza, Egypt, during the flowering stage (September 2016). It was identified by Dr. Trease Labib, former specialist of plant Taxonomy, El Orman Botanical Garden, Giza, Egypt. Voucher specimen (No.000105CC @ 05–01–05-01) was deposited at the herbarium of El Orman Botanical Garden, Giza, Egypt.

### Extraction and isolation

Air dried *C. quadrifidus* leaves (2 kg) were extracted by reflux with 80% aqueous MeOH (5 L) at law temperature (40 °C) for 4 h. The filtrated aqueous MeOH was evaporated under reduced pressure and law temperature to afford 105 g of dry crude extract. The dry residue was suspended in 300 mL H_2_O and successively fractionated with dichloromethane and ethyl acetate by liquid extraction (3 × 300 mL). After evaporation of each solvent, a total of 35, 20 and 45 g of dichloromethane (DCMF), ethyl acetate and aqueous extract dry residue were obtained respectively. By application of the three fractions on TLC and spraying with *p*-anisaldehyde / sulphuric acid reagent which is characteristic for terpenoidal compounds [5], it was found that the DCMF is rich in terpenoidal compounds than other two fractions. Therefore the DCMF was used for further isolation of terpenoidal compounds. It was applied on normal phase silica gel column (800 g × 1000 × 7 cm) and eluted with gradients of n-hexane-ethyl acetate (8:2: up to 2:8). Thirty -five fractions of 250 mL each were collected and combined into five major fractions on the basis of their TLC. F-1 was found to be rich in fatty substance and contains traces of terpenoidal compounds. F-2 (1.3 g) was chromatographed on successive silica gel column using n-hexane-ethyl acetate mixture (4:6) as eluent to afford pure sample of compound 1 (19 mg). F-3 (2.5 g) was fractionated on silica gel column and eluted with n- hexane- ethyl acetate mixture (3: 7) giving two main subfractions, each one contains crude sample of compound 2 and 3. For final purification of each one, they subjected on sephadex LH-20 column and eluted with MeOH to afford chromatographically pure samples of compounds 2 (20 mg) and 3 (25 mg). F-4 (3.4 g) was fractionated on silica gel column using DCM-MeOH (100:0 to 90–10) as eluent to give a fraction containing mixture of two compound which further purified using prep-TLC and DCM-MeOH (95:5) for development to yield pure samples of compound 4 (11 mg) and 5 (8 mg). It was found that F-5 (0.5 g) contains a complex mixture of minor compounds, so it is difficult to isolate them. Purity of the isolated compounds was established on the bases of their appearance under UV-254 and behaviour towards *p*-anisaldehyde/sulphuric acid spray reagent on TLC.

### Antibacterial activity

#### Materials

Gram positive bacteria;*Staphylococcus aureus* (RCMB010010) and *Bacillus subtilis* (RCMB 015 (1) NRRL B_−_ 543) and Gram negative bacteria;*Salmonella typhimurium* (RCMB 006 (1) ATCC 14028) and *Escherichia coli* (RCMB 010052 ATCC 25955) were supplied from the Regional Center for Mycology and Biotechnology (RCMB), Al-Azhar University, Cairo, Egypt. Müller-Hinton Agar (Sigma-Aldrich Company, USA); ampicillin (October pharma, Egypt); gentamycin (Garamycin, MUP for Schering-Plough, Egypt); and Triphenyltetrazolium chloride (Sigma–Aldrich, Chemical Company USA) were used for antimicrobial evaluation.

#### Susceptibility test

The antibacterial activity of the isolated compounds was investigated using agar well diffusion method [6]. Gram negative and positive bacteria (two of each) were used. Muller Hinton agar was used for the bacterial growth (45 ± 2 °C). The inoculum was culture of each bacterial species in the 20 mL Muller Hinton agar diluted in the same medium to a final concentration of approximately 1 × 108 CFU/mL (0.5 NTU – McFarland scale). After that it poured into sterile Petri dish and left until complete solidification and Wells were made using 8 mm diameter of sterile cork borer. The initial solution of the tested compounds were prepared by dissolving 10 mg in 1 mL dimethyl sulfoxide to obtain concentration of 10 mg/mL and then 100 μg/mL was added to each well. Ampicillin and gentamycin (5 mg/mL) as anti-bacterial controls and dimethyl sulfoxide control were added into the wells, separately. Plates were incubated at 37 °C for 24 h. The antibacterial activity of the compounds was determined by measuring the diameter of clear zone around the well [6]. Three replicates were maintained for each experiment.

#### Determination of minimum inhibitory concentration (MIC)

MIC of the pure compounds (1–5) was measured using microtiter plate dilution method [7]. 2-fold serial dilutions of the compounds were carried out in 100 μL nutrient broth to reach concentrations from 1000 to 4.7 μg/mL then the plates were incubated overnight at 37 °C. MIC was determined as the lowest concentration of compounds with no visible growth [8].

#### Statistical analysis

Data was analysed using one-way Analysis of Variance (ANOVA) followed by Tukey-Kramer Multiple Comparisons Test. The experimental results were expressed as a mean, ± Standard deviation (SD). The difference between groups were considered significant when *p* < 0.001. All analyses were performed using (GraphPad InStat®, version 3, USA) software.

## Results

### Identification of the isolated compounds

**Fig. 1:**
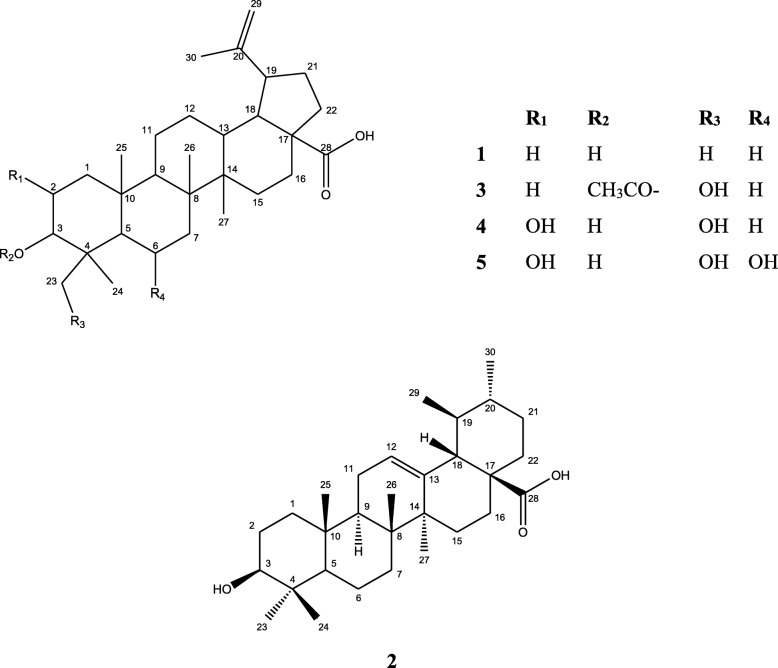
Structures of the isolated compounds 1–5 from DCMF of *C. quadrifidus* leaves

On the basis of their chromatographic properties including their appearance as violet color with *p-*anisaldehyde spray reagent, as well as spectral data (Table 1), it was found that the main skeleton of the compounds 1 and 3–5 (Fig. 1) belongs to lupane type [9].

**Table 1:** The ^1^H-NMR (600 MHz) and ^13^C-NMR (150 MHz) data of compound 1 and 3–5 (in DMSO); *δ* in ppm; *J* in Hz

Position	1		3		4		5	
	*δ*C	*δ*H	*δ*C	*δ*H	*δ*C	*δ*H	*δ*C	*δ*H
1	38.84		38.20		39.38		40.75	
2	27.61		27.55		67.43		67.66	
3	77.14	3.39 (1 H, brs)	82.52	3.83 (1 H, s)	77.30	3.62 (1 H, brs)	82.65	3.62 (1 H, brs)
4	38.93		38.87		38.87		40.40	
5	55.25		55.17		55.87		55.87	
6	18.42		18.43		18.46		77.35	2.13 (1 H, m)
7	34.37		34.45		36.82		40.40	
8	40.66		40.49		40.40		39.38	
9	50.36		50.32		48.97		48.97	
10	37.18		37.22		38.02		38.02	
11	20.91		20.91		21.54		22.61	
12	25.52		25.47		25.56		23.81	
13	38.04		38.04		38.95		38.95	
14	42.45		42.45		42.48		42.48	
15	30.55		30.55		31.81		30.85	
16	32.16		32.16		32.17		32.17	
17	55.94		55.84		55.87		55.87	
18	47.07		47.02		47.07		47.07	
19	49.07		48.97		48.93		48.97	
20	150.82		150.67		150.37		150.60	
21	29.66		29.53		29.09		29.28	
22	36.80		36.70		36.81		36.81	
23	28.55	1.32 (3 H, s, Me)	63.21	3.70 (2 H, s)	62.87	3.69 (2 H, s)	63.26	3.70 (2 H, s)
24	16.17	1.11 (3 H, s, Me)	16.42	1.32 (3 H, s, Me)	17.35	1.32 (3 H, s, Me)	17.35	1.38 (3 H, s, Me)
25	16.27	0.87 (3 H, s, Me)	17.37	0.91 (3 H, s, Me)	17.58	0.81 (3 H, s, Me)	17.58	0.76 (3 H, s, Me)
26	16.41	0.77 (3 H,s, Me)	17.60	1.07 (3 H,s, Me)	16.48	0.96 (3 H,s, Me)	18.46	1.08 (3 H,s, Me)
27	14.84	0.93 (3 H, s, Me)	16.14	1.08 (3 H, s, Me)	14.80	0.71 (3 H, s, Me)	14.80	0.82 (3 H, s, Me)
28	177.72	12.03 (1 H, brs)	177.60	12.03 (1 H, brs)	177.98	12.07 (1 H, brs)	177.75	12.04 (1 H, brs)
29	110.11	4.69 (1 H, brs, H-29^a^) 4.56 (1 H, brs, H-29^b^)	109.96	4.69 (1 H, brs, H-29^a^) 4.57 (1 H, brs, H-29^b^)	109.21	4.69 (1 H, brs, H-29^a^) 4.57 (1 H, brs, H-29^b^)	110.10	4.69 (1 H, brs, H-29^a^) 4.57 (1 H, brs, H-29^b^)
30	19.39	1.65 (3 H,s, Me)	19.39	1.65 (3 H,s, Me)	19.40	1.65 (3 H,s, Me)	19.40	1.65 (3 H,s, Me)
31			172.53					
32			21.55	1.91 (3 H,s, Me)				

^a,b^ represent the two geminal protons on C_29_

Compound 1 was obtained as white crystals (19 mg), *R*_*f*_-value 0.80 (S_1_); IR spectrum clearly showed the presence of carboxylic and hydroxyl groups at 3500–2500 cm^− 1^ and broad band at≈ 3400 cm^− 1^ respectively, characteristic for lupane nucleus [9, 10]. The ^1^H-NMR spectrum of 1 (Table 1) suggested that its structure may be betulinic acid based on the presence of six sharp singlets appearing at δ1.65, 1.32, 1.11, 0.93, 0.87 and 0.77 ascribed to the protons of six tertiary methyl groups (H-30, H-23, H-24, H-27, H- 25 and H-26, respectively) in addition to one proton multiplet at δ 3.39 for the hydroxyl function at C-3 and two doublets at δ 4.56 (1H, d) and at δ 4.69 (1H, d) respectively, characteristic for exomethylene group [11]. The structure was established through its APT ^13^C-NMR data (Table 1) which revealed the presence of carbon signal at δ77.14 confirming the hydroxylation of C-3 as well as two olefinic carbons resonating at *δ* 150.82 and 110.11 ppm for the exomethylene group. Moreover the carboxylic group was established form the carbon signal at *δ* 177.72 [12]. From the above data and in comparison to the previously available data [13, 14]; compound 1 was identified as betulinic acid.

Compound 3 was isolated as white crystals (25 mg), R_*f*_-value 0.70 (S1). The spectral data of 3 were very close to 1 which gave evidence that its structure is betulinic acid derivative. ^1^H-NMR of 3 (Table 1) was the same as that of 1 except showing a signal at δ 1.91 (3H, s) that is characteristic for an acetyl group which was confirmed from^13^C-NMR (Table 1) by the presence of carbon signals at δ21.55 and 172.5 for methyl and ester carbonyl of acetate moiety. Also, the position of acetyl group at C-3 was established from the downfield shift of H-3 at δ3.83 and C-3 at δ 82.52 [15]. Moreover the presence of singlet signal integrated for 2H, at δ 3.70 with carbon signal at 63.21 together with the comparison to published data [16], gave evidence that methyl group of C-23 was replaced by hydroxyl methylene group. Based on the previous and literature data, compound 3 was identified as 3-acetyl-23-hydroxy betulinic acid.

Compound 4 was obtained as white crystals (11 mg) with R_*f*_-value 0.65 (S_1_). It was expected to have the same skeleton of 1 and 3 through comparison of their spectral data. The ^13^C-NMR spectra of 4 indicated that C-2 and C-23 are downfield at δ 67.43 and 62.87 respectively, suggesting their substitution by a hydroxyl group [10, 11]. The remaining assignments of ^1^H and ^13^C-NMR data were in a good agreement with previously published data [17] so the structure of 4 was established as 2, 23-dihydroxy betulinic acid.

Compound 5 is white amorphous powder (10 mg) having R_f_-value of 0.61 (S_1_). Its ^13^C-NMR spectra almost resemble that of 4 with the exception of the downfield shift of C-6 at δ C 77.35 (18.46 in case of 4) which gave evidence that the C-6 is hydroxylated. Further proof of structure was achieved from HMBC spectrum which showed the most important correlations between H-3 (δ 3.62) with C-2 (δ 67.66), C-23 (δ 82.65) and H-6 (δ 2.13) with C-7 (δ 40.4), C-5 (δ 55.87). All remaining correlations supported that the structure of 5 is 2, 6, 23-trihydroxy betulinic acid. All data were in a good agreement with data published before [11, 18].

Compound 2 was obtained as white needles (21 mg); R_f_-value 0.77 (S1). IR νmax (cm^− 1^): 3422, 2927, 1693.^1^H NMR (600 MHz, DMSO-*d*_*6*_), δ ppm 0.80, 0.98, 1.04, 1.08, 1.10 and 1.64 (6 s, 18H, all tertiary –CH3), 3.39 (1 H, brs, H-3), 5.13 (1 H, brs, H-12), 2.22 (1 H, t, *J* = 10.82 Hz, H-15), 2.75 (1 H, d, *J* = 9.70 Hz, H-18); ^13^C NMR (125 MHz, DMSO): δ = 178.72 (C-28, COOH), 38.06 (C-20), 19.41 (C-29), 77.29 (C-3), 47.29 (C- 17), 55.37 (C-5), 50.84 (C-9), 55.26 (C-18), 38.06 (C-19), 42.10 (C-14), 40.72 (C-8), 40.6 (C-4), 39.57 (C-1), 138.64 (C-13), 38.7 (C-10), 36.99 (C-22), 34.4 (C-7), 27.63 (C-16), 32.18 (C-21), 28.73 (C-23), 29.68 (C-2), 30.57 (C-15), 125.04 (C-12), 25.55 (C-11), 21.55 (C-30), 20.94 (C-6), 17.48 (C-26), 15.70 (C-25), 17.37 (C-24), 23.74 (C-27). Based on its spectral data as well as comparison with authentic sample and previous data, compound 2 was identified as ursolic acid [19].

### Antibacterial activity

The Antibacterial activity of compounds (1–5) isolated from the DCMF of *C. quadrifidus* leaves was performed against four bacterial strains including two gram negative *S. typhimurium* and *E.coli* and two gram positive *S. aureus* and B subtilis (Tables 2 and 3). Compounds 4 and 5 were the most active against *S. typhimurium* (MIC = 125 μg/mL) while compound 1 and 2 showed moderate activity against the same bacteria (MIC = 625 μg/mL). Furthermore compounds 4 and 5 exhibits a strong activity against *E.coli* (MIC = 312 μg/mL) and compound 3 showed moderate activity toward it (MIC = 625 μg/mL). Moreover compound 3 and 4 showed strong activity against *S. aureus* (MIC = 312 μg/ mL) and compound 1 showed moderate activity against it (MIC = 625 μg/mL). In addition the B subtilis was moderately inhibited by compound 3, 4 and 5 (MIC = 625 μg/mL). The standard drugs used in this study were gentamycin and ampicillin for antibacterial and the antibacterial activities of compound 4 and 5 against *S. typhimurium* were slightly less than the activity of gentamycin.

**Table 2:** Antibacterial activity of the pure compounds 1–5

Bacteria	Compound					Positive control
	1	2	3	4	5	
Gram + ve						Ampicillin
*S.aureus*	11.03 ± 0.45	9.37 ± 0.32	13.6 ± 0.18	14.5 ± 0.45	10.03 ± 0.95	24.13 ± 1.21
*B.subtilis*	10.17 ± 0.26	10.65 ± 0.46	12.5 ± 0.44	11.87 ± 0.65	11.18 ± 0.51	25.97 ± 0.95
Gram - ve						Gentamycin
*S.typhimurium*	8.70 ± 0.79	9.78 ± 0.65	11.09 ± 0.13	15.02 ± 0.95	13.89 ± 0.45	16.97 ± 0.95
*E. coli*	12.8 ± 0.71	11.9 ± 0.35	16.75 ± 0.65	17.95 ± 0.35	18.7 ± 0.7	30.03 ± 1.05

Results were expressed as mean IZ ± S.D

**Table 3:** Minimum inhibitory concentration (MIC) as μg/mL for 1–5

Bacteria	Compound					Positive control
	1	2	3	4	5	
Gram + ve						Ampicillin
*S.aureus*	625	2350	312	312	2500	90
*B.subtilis*	1250	5000	625	625	625	65
Gram - ve						Gentamycin
*S.typhimurium*	625	625	212.5	125	125	100
*E. coli*	1250	1250	625	312	312	65

MIC values expressed as μg/mL

## Discussion

All isolated compounds (1–5) were previously identified from the literature but they are isolated here for the first time from the leaves of *C. quadrifidus* as well as we focused on their antibacterial activity since many studies were concentrated for search for new antibacterial agents from natural sources due to the resistance of human pathogenic microorganisms to the major antibiotics [20]. Triterpenes are known to display significant antimicrobial properties [21, 22]. Terpenoidal compounds are used in the treatment of bacterial infections due to their lipophilic properties which allow them to be easily interacting with the bacterial wall, interfering with the biosynthesis of its components as well as they can penetrate the bacterial cell and may also interfere with protein synthesis and DNA replication and repair mechanisms. Our study showed that there were differences between the antimicrobial activities of the isolated compounds which may be due to the difference in the substation groups as well as the its position. It was shown from the biological assay results that the hydroxyl group at the position C- 23 [23] makes an important contribution to the expression of activity in compound 3, 4 and 5. Also the difference in the structure between the betulinic acid, its derivatives (compounds 1, 3–5) and ursolic acid may affect the difference in activity [24].

## Conclusion

The current study resulted in the identification of antibacterial triterpenoidal compounds from DCMF of *C. quadrifidus* leaves for first time. In our future study we will carry out more experimental and clinical trials to establish this finding for the development of new antibacterial natural drugs.

## Abbreviations

DCM: Dichloromethane
DCMF: Dichloromethane fraction
F: Fraction
IZ: Inhibition zone
MIC: Minimum inhibitory concentration
Prep TLC: Preparative thin layer chromatography
S: Solvent system
SD: Standard deviation
SPSS: Statistical Package for Social Sciences
